# Complete Genome Sequence of Klebsiella pneumoniae Bacteriophage KpS110, Encoding Five Tail-Associated Proteins with Putative Polysaccharide Depolymerase Domains

**DOI:** 10.1128/mra.00153-23

**Published:** 2023-04-18

**Authors:** Nikolay V. Volozhantsev, Vladimir V. Verevkin, Valentina M. Krasilnikova, Angelina A. Kislichkina, Anastasia V. Popova

**Affiliations:** a State Research Center for Applied Microbiology and Biotechnology, Obolensk, Russia; Queens College Department of Biology

## Abstract

We report the genome sequence of bacteriophage KpS110, which infects Klebsiella pneumoniae, a multidrug-resistant encapsulated bacterium that causes severe community-acquired and hospital-acquired infections. The phage genome is 156,801 bp, with 201 open reading frames. KpS110 is most closely related to phages of the family *Ackermannviridae* at the genome and proteome levels.

## ANNOUNCEMENT

Klebsiella pneumoniae, a multidrug-resistant, encapsulated, Gram-negative bacterium, causes severe community-acquired and hospital-acquired infections, such as septicemia, pneumonia, urinary tract infections, surgical site infections, and catheter-related infections (1, 2). The high antibiotic resistance of K. pneumoniae, the ability to acquire resistance, and biofilm formation complicate the treatment of infections caused by this pathogen. K. pneumoniae is a critical priority pathogen to guide the discovery, research, and development of new antibacterial agents (3) and is a potential target for bacteriophage therapy.

In this study, we report the genome sequence of the bacteriophage (phage) vB_KpnM_KpS110 (hereafter KpS110). The phage was isolated in 2016 from raw sewage samples in Serpukhov district, Moscow region, Russia, on the bacterial lawn of the multidrug-resistant K. pneumoniae strain KPB1667 (capsular type K47), which had been previously isolated from a hospitalized patient (4). The phage and host strain were deposited in the State Collection of Pathogenic Microorganisms and Cell Cultures (SCPM-Obolensk) (https://obolensk.org/science/state-coll-microorg) under accession numbers Ph-126 and B-8043, respectively.

A high-titer phage lysate was prepared by overnight phage propagation in a log-phase culture of the strain KPB1667 in LB medium at 37°C with aeration. KpS110 DNA was isolated by phenol-chloroform extraction as described previously (5). The DNA library was prepared using the Ion Xpress Plus fragment library kit (Life Technologies, Inc.) and then size selected with 2% E-Gel SizeSelect (Invitrogen). The library was amplified on the OneTouch 2 system with the Ion PGM Template OT2 400 kit (Life Technologies, Inc.) and sequenced on an Ion Torrent PGM instrument using the 314 Chip kit v2 and Ion PGM sequencing kit (Life Technologies, Inc.). The resultant 42,075 reads, with an average length of 150 bp and 35× coverage, were assembled into a closed contig with Newbler v2.9 and corrected with DNASTAR SeqMan NGen software. Protein-coding genes were predicted using the GeneMarkS software tool (6), RAST automated annotation engine (7), and NCBI BLAST algorithms (8). Additionally, we used the HHpred interactive server for protein homology detection and structure prediction (9). All tools were run with default parameters.

Phage KpS110 has a 156,801-bp linear double-stranded DNA genome, with a GC content of 46.14%. Within the phage genome, 201 protein-coding sequences (CDSs) and 6 tRNA genes were predicted. Putative functions were assigned to 74 phage-encoded proteins, and the rest were annotated as hypothetical proteins. We could not assign phage genome ends. Nevertheless, the genome circularized upon assembly, indicating a complete genome and a possible headful DNA packaging strategy. Moreover, the *terL* gene of KpS110 shows high homology to those of phages that use a headful packaging mechanism (10).

The phage KpS110 was assigned to the family *Ackermannviridae*, genus *Taipeivirus*, whose genomes are characterized by the presence of several genes encoding tail spike proteins (TSPs) with polysaccharide-depolymerizing properties (11). We identified 5 CDSs encoding putative TSPs (Table 1), which are grouped into a 11,886-bp cluster. HHpred analysis showed that the products of the aforementioned genes have structural homology to proteins containing β-helical regions and hydrolase domains, which are characteristics of structural phage depolymerases.

**TABLE 1:** Phage KpS110 genes encoding proteins with putative polysaccharide depolymerase domains

CDS	Product	GenBank accession no.	Closest homolog in the PDB (E value)	Catalytic domain	PDB DOI no. (ref.)
kps110_112	TSP	AUV59228.1	PhiAB6 tail spike (0.000071)	Hydrolase; β-helix, superhelical trimer	10.2210/pdb5JS4/pdb (12)
kps110_113	Hypothetical protein	AUV59229.1	Bifidobacterium longum lacto-*N*-biosidase (2.7e−27)	Hydrolase; β-helix	10.2210/pdb5GQF/pdb (13)
kps110_114	TSP	AUV59230.1	Phage CBA120 tail spike (7.1e−29)	Hydrolase; β-helix, superhelical trimer	10.2210/pdb5W6H/pdb (14)
kps110_116	Tail fiber protein	AUV59232.1	Phage phi92 gp150 (2e-54)	Hydrolase/colanidase; β helix	10.2210/pdb6E0V/pdb
kps110_118	Hypothetical protein	AUV59234.1	Phage CBA120 tail spike (4.5e−24)	Putative endoglycosidase; parallel β-helix	10.2210/pdb6NW9/pdb (15)

### Data availability.

The phage whole-genome sequence was deposited in GenBank under accession number MG770379. The raw sequencing reads are available in the SRA under SRA accession number SRR23681980 and BioProject accession number PRJNA938985.
